# Efficacy of endoscopic submucosal tunnel dissection in the management of a large esophageal cavernous hemangioma

**DOI:** 10.1055/a-2421-6218

**Published:** 2024-10-16

**Authors:** Guang Yang, Silin Huang, Suhuan Liao, Yang Lv, Wei Gong, Qiuping Qiu, Nan Liu

**Affiliations:** 1 Department of Gastroenterology, South China Hospital, Medical School, Shenzhen University, Shenzhen, China; 2 559569 Department of Gastroenterology, Shenzhen Hospital of Southern Medical University, Shenzhen, China; 3 Institute of Environment and Health, South China Hospital of Shenzhen University, Shenzhen, China

A 44-year-old man presented with symptoms of gastroesophageal reflux disease and dysphagia. Gastroscopy revealed a 3-cm, half-circumferential, bluish-purple esophageal mass located in the mid-esophageal region ( Fig. 1 **a** ). Computed tomography revealed a soft tissue nodule causing significant stenosis of the esophageal lumen. Endoscopic ultrasound confirmed a well-demarcated, moderately hyperechoic submucosal lesion, characteristic of an esophageal cavernous hemangioma ( Fig. 1 **b** ). Subsequent to a detailed consultation, endoscopic submucosal tunnel dissection (ESTD) was undertaken ( Video 1 ).

**Fig. 1:**
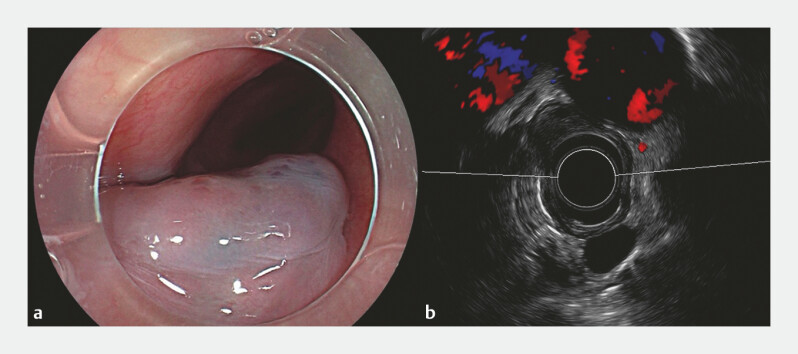
Colonoscopy and endoscopic ultrasound. **a** Gastroscopy revealed a 3-cm, half-circumferential, bluish-purple esophageal mass located in the mid-esophageal region. **b** Endoscopic ultrasound confirmed a well-demarcated, moderately hyperechoic lesion within the submucosal layer.

Efficacy of endoscopic submucosal tunnel dissection for the management of a large esophageal cavernous hemangioma.Video 1

Using a hybrid knife (Erbe Elektromedizin GmbH, Tübingen, Germany), saline mixed with indigo carmine was injected 0.5 cm proximal to the lesion, followed by a 1.5-cm transverse incision to create a submucosal tunnel extending 1 cm distally ( Fig. 2 **a** ). A significant presence of perforating vessels was observed in the submucosal layer, prompting the use of soft electrocoagulation for meticulous hemostasis ( Fig. 2 **b** ). An additional 1.5-cm incision was made distally. Incremental dissection along both tunnel margins was performed, achieving complete en bloc resection with a 0.5-cm margin from the tumorʼs edge. Electrocoagulation was applied to exposed vessels to control bleeding, with no damage to the muscular layer ( Fig. 2 **c** ). The procedure was completed in 30 minutes without complications, including perforation, hemorrhage, or fever. Histopathological analysis confirmed esophageal cavernous hemangioma ( Fig. 2 **d** ). The patient was discharged on postoperative day four and remained symptom-free during 12 months of follow-up.

**Fig. 2:**
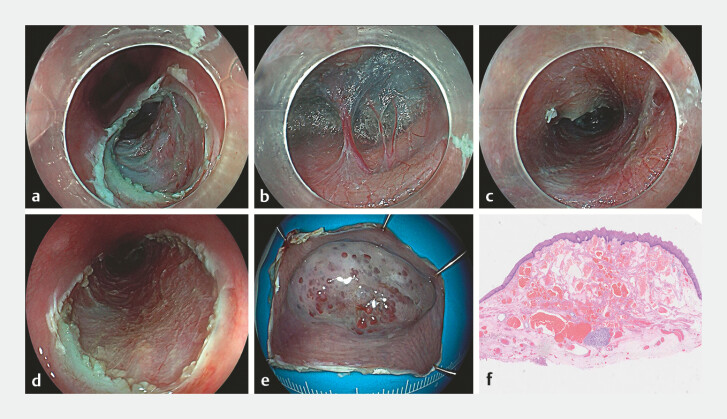
Endoscopic submucosal tunnel dissection. **a** A transverse incision was made on the oral side of the lesion to establish the tunnel entry point. **b** The submucosal layer revealed a notable abundance of perforating vessels. **c** A submucosal tunnel was meticulously fashioned, extending 1 cm distally from the incision. **d** Postoperative wound. **e** The tumor was successfully resected in its entirety. **f** Histopathological examination confirmed the diagnosis of esophageal cavernous hemangioma.

Esophageal cavernous hemangioma is a rare benign tumor 1 , with management options for asymptomatic cases typically involving observation, whereas symptomatic cases may necessitate intervention. Treatment approaches include esophageal resection, tumor dissection, endoscopic sclerotherapy, and laser therapy 2 . Endoscopic submucosal dissection has been utilized for esophageal hemangiomas 3 4 , and our case illustrates that ESTD enhances submucosal visualization and expedites dissection. This represents the first successful en bloc resection of a symptomatic esophageal cavernous hemangioma via ESTD.

Endoscopy_UCTN_Code_TTT_1AO_2AC

## Notes

Endoscopy E-Videos https://eref.thieme.de/e-videos
*E-Videos* is an open access online section of the journal *Endoscopy* , reporting on interesting cases and new techniques in gastroenterological endoscopy. All papers include a high-quality video and are published with a Creative Commons CC-BY license. Endoscopy E-Videos qualify for HINARI discounts and waivers and eligibility is automatically checked during the submission process. We grant 100% waivers to articles whose corresponding authors are based in Group A countries and 50% waivers to those who are based in Group B countries as classified by Research4Life (see: https://www.research4life.org/access/eligibility/ ). This section has its own submission website at https://mc.manuscriptcentral.com/e-videos .

## References

[1] Araki K Obno S Egashira A Esophageal hemangiona; a case report and review of the literature Hepatogastroenterology 1999 46 3148 3154 10626176

[2] Rodrigues-Pinto E Pereira P Macedo G Bluish discoloration of the esophagus: cavernous hemangioma of the pharynx and larynx with esophageal involvement Endoscopy 2015 47 E213 E214 10.1055/s-0034-1391823 26062154

[3] Zhu Z Wang L Yin J Endoscopic submucosal dissection for a symptomatic cervical esophageal cavernous hemangioma Endoscopy 2022 54 E604 E605 10.1055/a-1694-3217 35081642

[4] Chedgy FJ Bhattacharyya R Bhandari P Endoscopic submucosal dissection for symptomatic esophageal cavernous hemangioma Gastrointest Endosc 2015 81 998 10.1016/j.gie.2014.10.023 25484322

